# Prevalence and Associations of Psychological Distress, HIV Infection and HIV Care Service Utilization in East Zimbabwe

**DOI:** 10.1007/s10461-017-1705-x

**Published:** 2017-02-13

**Authors:** Malebogo Tlhajoane, Jeffrey W. Eaton, Albert Takaruza, Rebecca Rhead, Rufurwokuda Maswera, Nadine Schur, Lorraine Sherr, Constance Nyamukapa, Simon Gregson

**Affiliations:** 1 Department for Infectious Disease Epidemiology, Imperial College London, Norfolk Place, London, W2 1PG UK; 2 Biomedical Research and Training Institute, Harare, Zimbabwe; 3 Institute of Epidemiology and Health, University College London, London, UK

**Keywords:** Mental health, Antiretroviral therapy, HIV/AIDS, Adherence, Treatment cascade

## Abstract

The correlation between mental health and sexual risk behaviours for HIV infection remains largely unknown in low and middle income settings. The present study determined the prevalence of psychological distress (PD) in a sub-Saharan African population with a generalized HIV epidemic, and investigated associations with HIV acquisition risk and uptake of HIV services using data from a cross-sectional survey of 13,252 adults. PD was measured using the Shona Symptom Questionnaire. Logistic regression was used to measure associations between PD and hypothesized covariates. The prevalence of PD was 4.5% (95% CI 3.9–5.1%) among men, and 12.9% (95% CI 12.2–13.6%) among women. PD was associated with sexual risk behaviours for HIV infection and HIV-infected individuals were more likely to suffer from PD. Amongst those initiated on anti-retroviral therapy, individuals with PD were less likely to adhere to treatment (91 vs. 96%; age- and site-type-adjusted odds ratio = 0.38; 95% CI 0.15, 0.99). Integrated HIV and mental health services may enhance HIV care and treatment outcomes in high HIV-prevalence populations in sub-Saharan Africa.

## Introduction

Depression is the leading cause of disability worldwide, affecting an estimated 350 million people [1]. Despite the large burden of mental and behavioural disorders across the world, resources for mental health care remain limited, especially in low and middle income countries (LMIC). The World Health Organization (WHO) estimates that between 76 and 85% of individuals with a severe psychological disorder in LMIC receive no treatment [2]. In addition, the total population (in millions) per mental health outpatient facility was 3.31 in the WHO Africa region compared to 0.08 in Europe [3]. By 2030, depression alone is predicted to be the highest contributor to the global burden of disease [4].

While notable declines in the prevalence and incidence of HIV have been reported in Zimbabwe over the past decade, an estimated 1.4 million people were living with HIV in 2015 [5, 6]. Since the introduction of the public sector antiretroviral therapy (ART) programme in 2004, national ART coverage has steadily increased from 30% in 2010 to 62% in 2015 [6]. Although the availability of HIV care and treatment services has increased, the provision of mental healthcare services remains limited. The 2010 Global Burden of Disease (GBD) study estimated that major depressive disorders were among one of the top five causes of years lived with a non-fatal disability (YLD) in Zimbabwe [7]. Previous estimates of psychiatric morbidity among people living with HIV (PLHIV) within the country have ranged from 42 to 63% [8, 9]. Despite this, there were only 0.02 mental health outpatient facilities, and 0.06 specialist psychiatrists per 100 000 members of the population in 2011 [10].

As the burden of depressive disorders continues to grow, a better understanding of the intersection between mental health and HIV is of increased importance, particularly in high HIV-prevalence settings in sub-Saharan-Africa [11]. Individuals with poor mental health may be at elevated risk of engaging in risky sexual practices; thereby, increasing their likelihood of acquiring or transmitting HIV infection [12, 13]. Psychological distress (PD) can be defined as a general term for a state of emotional suffering that can be associated with common mental disorders [14]. Among key HIV-infected groups in developed countries, PD, including depression, anxiety and post-traumatic stress disorder, has been associated with high-risk sexual behaviours including prostitution and inconsistent condom use [15–19]. Whilst a number of studies have been conducted in low income countries, the great majority of these have been small-scale studies conducted in HIV clinics or psychiatric settings [13, 20–24].

In cross-sectional studies conducted amongst attendees at HIV care facilities in sub-Saharan Africa, psychological distress has been associated with treatment interruptions and sub-optimal adherence to ART, increasing the potential for virological failure, poor disease outcomes and further spread of infection [20–25]. However, the preponderance of evidence from these populations may create bias towards health seeking individuals whose symptoms are more severe such that they warrant institution-based care. Large population-based studies are therefore needed in low-income, high HIV-prevalence countries to establish the nature and extent of interactions between poor mental health, HIV risk, uptake of HIV services and adherence to ART in these settings. In this study, we aim to: (i) determine the prevalence of psychological distress in a general population sample in eastern Zimbabwe, a low-income country suffering hyper-endemic HIV; (ii) describe patterns of association between psychological distress and hypothesized socio-demographic, behavioural and clinical risk factors based on a theoretical framework; (iii) assess the relationship between psychological distress and sexual risk behaviours that may increase the likelihood of HIV acquisition and transmission; and (iv) evaluate associations between psychological distress and use of HIV testing services, receipt of cotrimoxazole, and uptake and adherence to ART within the population.

## Methods

### Theoretical Framework

**Fig. 1:**
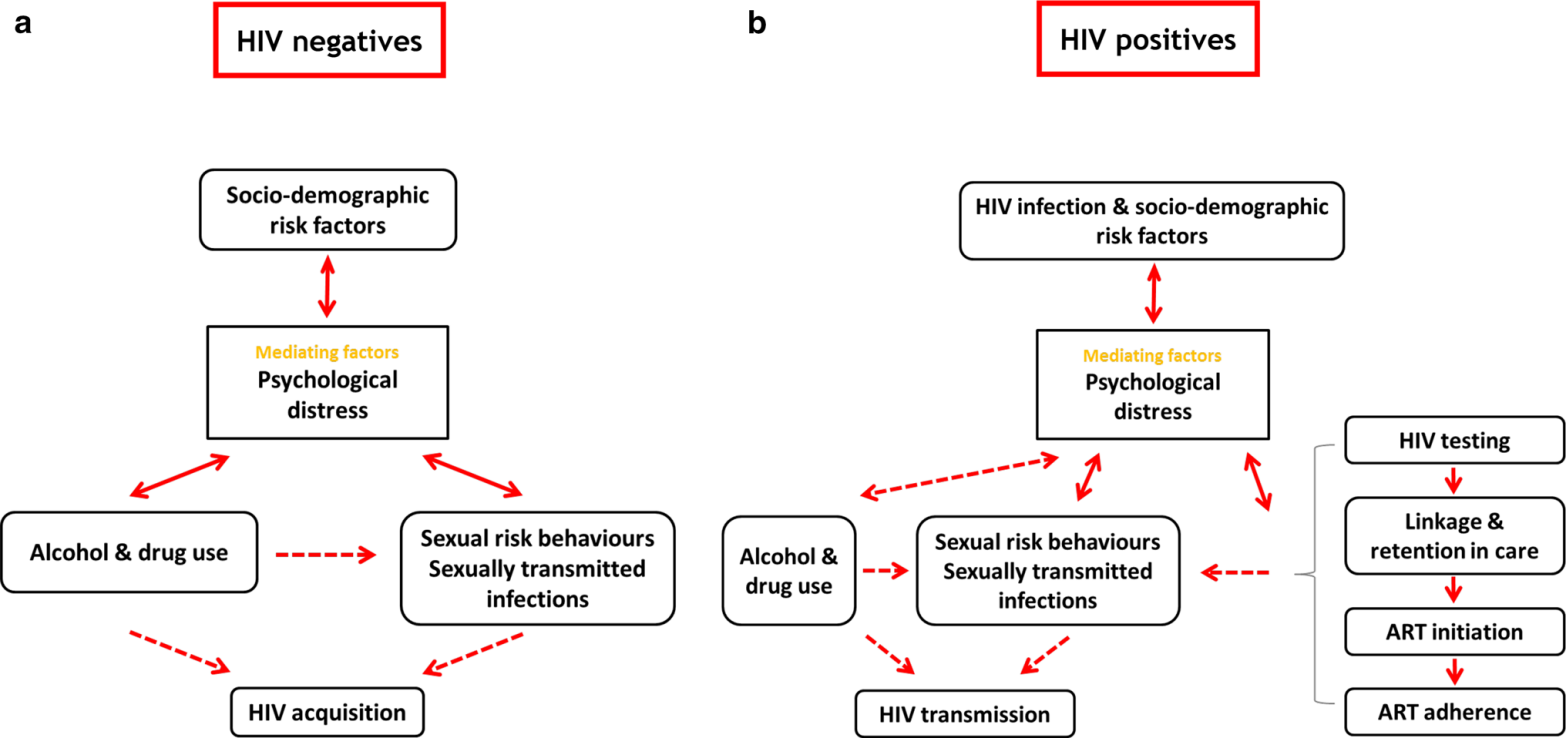
Theoretical framework for the causes and consequences of psychological distress in HIV-uninfected (**a**) and HIV-infected (**b**) individuals in a population subject to hyper-endemic HIV

A theoretical framework was developed from published literature to guide the analysis of the inter-relationships between psychological distress, HIV infection, and use of HIV services in the study population. Among individuals not infected with HIV (Fig. 1a), we hypothesised that background and socio-demographic factors, including site type, food security, sex, age, education, marital status, religion, employment, and social support, would be associated with psychological distress. Psychological distress, in turn, was hypothesised to have bi-directional relationships with alcohol and recreational drug use, and with high-risk sexual behaviours in populations subject to hyper-endemic HIV. Directly and, through their effects on other sexually transmitted infections, these behaviours are likely to increase the risk of HIV acquisition.

For already HIV-infected individuals, similar associations were hypothesised to occur, potentially leading to a link between psychological distress and onward transmission of infection to greater numbers of sexual partners. In addition, psychological distress could have an impact on the likelihood of taking up HIV testing, linkage to care, retention in care, initiation on ART, and adherence to ART. Such effects can subsequently impact on HIV transmission where, for example, poor adherence may result in viral resistance and an elevated risk of transmission, as well as transmission of resistant strains. In some instances, reverse effects may also occur (e.g. people failing to adhere to ART can experience psychological distress as a consequence).

### Study Population and Data Collection

Data for the analysis were taken from the Manicaland HIV/STD Prevention Project, a longitudinal general population open cohort survey established in 1998 in eastern Zimbabwe [5, 26]. The study area is comprised of 12 sites (2 small towns, 4 agricultural estates, 2 roadside trading centres, and 4 subsistence farming areas) within the Manicaland province of Zimbabwe. A preliminary census of all households at each site in the province was conducted in 1998 to identify individuals eligible for inclusion into the cohort. Full details of the sampling methods have been previously described [5]. In the current study, data were used from the fifth round of the survey in which 14,467 individuals aged 13–84 completed a face-to-face interview between July 2009 and August 2011 which included questions on psychological health for the first time. Collection of sexual behaviour data was done using an “informal confidential voting interview” procedure to reduce social desirability bias [27]. Dried blood spot samples were also collected from each respondent on the day of the interview for HIV testing [5]. Specimens were collected and air-dried prior to storage and tested for HIV antibodies using a dipstick dot-Enzyme Immunoassay. Confirmatory tests were conducted for all positive samples, as well as a 10% sample of negatives in the first few rounds of the study. In subsequent rounds, including round 5, confirmatory testing was done for sero-conversions only [5]. Full details of enumeration and HIV testing procedures of DBS samples for the Manicaland cohort have been described previously [5].

Amongst the respondents, 670 (4.6%) fell outside of the 15–54 age range and 545 (3.8%) had incomplete psychological health data. These individuals (n = 1215) were excluded from further analysis in the current study yielding a total sample size of 13,252.

### Measures and Procedures

#### Psychological Distress

For this analysis, a measure of psychological distress was derived from the Shona Symptom Questionnaire (SSQ). This questionnaire comprises a 14-item set of ‘yes’ or ‘no’ questions, and was developed and validated in Zimbabwe in 1997 with the aid of mental healthcare providers. The SSQ provides a case finding tool specific to the region [28], and examines psychological distress through somatic and psychological experiences over a period of time. In defining psychological distress, cut-off points were determined using validation data where the researchers found that suitable levels of sensitivity and specificity occurred at a cut-off point of 7/8 for the SSQ [28]. This cut-point generated a test sensitivity of 67%, specificity of 83% and a misclassification rate of 22%. In the current study, the key aim was to detect cases of psychological distress within a large, representative general population sample and thus a less conservative score of ≥7 was utilised to increase the sensitivity of the screening tool. At a cut-off score of 6/7, the SSQ yielded a sensitivity of 74%, specificity of 79% and a misclassification rate of 23%. All individuals with an SSQ score ≥7 were taken to have psychological distress and from this, a dichotomous variable (0/1) was created with individuals coded 1 being taken to suffer from psychological distress [28].

#### Risk Factors for Psychological Distress

For food security, a variable was used based on the date of interview and division of the year into quarters representing the early and late rainy seasons (October–December, and January–March, respectively), the immediate post-harvest period (April–June), and the subsequent dry season (July–September). Age was stratified into four groups (15–24, 25–34, 35–44 and 45–54 years). The highest level of educational attainment was captured using an ordered categorical variable where those who had not received formal education or had only received primary education were combined to form one group. Religious affiliation was categorized as traditional religion, Christian churches, spiritual religions, other religious groups, or no religious affiliation [29]. A dichotomous employment variable (unemployed/employed) was created. Social support was measured by two variables indicating membership within a well-functioning social group or a well-functioning church group [12].

High alcohol consumption was defined as reporting regular bar visits or ≥3 drinks per visit. Recreational drug use was based on self-reported injection, ingestion or smoking, and sexually transmitted infections were self-reported as experiencing pain, sores or a discharge in the genital region over the previous 12 months. HIV status was determined using the study DBS test result. For individuals without a study test result (n = 58), self-reported HIV status was used where available (n = 27). Among the respondents, 3.8% (n = 504) did not have complete data for all covariates of interest for analysing the hypothesized risk factors for psychological distress. These individuals were therefore excluded from this portion of the analysis.

#### Sexual Risk Behaviours for HIV Infection

Sexual risk behaviours for HIV infection were self-reported by survey participants who had started having sex (n = 10,377). Specific behaviours considered were having more than one sexual partner in the past 30 days; having concurrent sexual partnerships; inconsistent condom use while having a partner in a concurrent partnership; transactional sex; and sex work among women [30]. These behaviours have been shown previously to be associated with HIV infection [31, 32].

#### HIV Care Cascade

Uptake of HIV testing was measured dichotomously (ever tested vs. never tested) based on the self-reported number of times the respondent had received an HIV test in their lifetime. Collection of HIV test results was not taken into consideration. Further survey questions addressed uptake of HIV services and adherence to ART for individuals who reported having received a positive test result and who were positive by study DBS (n = 875). Receipt of cotrimoxazole prophylaxis was used as an indicator of having been initiated and retained in care for HIV-positive participants not yet on ART. Uptake of ART was assessed using a survey question on whether the respondent had ever taken drugs to stop HIV from causing AIDS. Those who reported receiving cotrimoxazole prophylaxis but not ART were excluded. Adherence to ART was assessed using survey questions on whether the respondent stopped taking antiretroviral drugs or sometimes forgets or doesn’t take drugs for other reasons. None of the women interviewed cited having received ART for prevention of vertical transmission as the reason for having stopped ART.

### Statistical Analysis

Summary statistics stratified by sex were computed to describe the study population and to examine the occurrence of psychological distress and co-variates. Binary logistic regression was used to test for associations between the hypothesised risk factors for poor mental health and psychological distress. Two multivariable models were built, the first adjusting for age only and the second adjusting for all variables with a p value ≤ 0.2 in the age-adjusted results, as well as age, site type and church grouping [33, 34].

In these models, for ordered categorical variables, the lowest group or the group that did not express the outcome of interest was used as the reference group; for nominal variables, the group with the largest population was used.

Logistic regression was also used to calculate site-type and age-adjusted odds ratios for associations between psychological distress and the sexual risk behaviour variables and between psychological distress and the uptake of HIV testing, separately, in HIV-uninfected and HIV-infected individuals. For HIV-infected individuals, site-type and age-adjusted odds ratios were calculated for receipt of cotrimoxazole prophylaxis (adjusted also for ART status), and for ART uptake. For HIV-infected individuals who had initiated ART, crude and site-type and age-adjusted odds ratios were calculated for adherence.

All data management and statistical analyses were performed using STATA 13.1 (StataCorp, College Station, TX, USA). Written informed consent was obtained from all study participants prior to the interview or provision of the blood sample.

## Results

13,252 respondents aged 15–54 years were interviewed in the Manicaland survey and had complete SSQ, psychological health data. The demographic characteristics of the study population are summarized in Table 1. HIV prevalence was 12.7% (648/5099) and 18.3% (1484/8122) for males and females, respectively, including self-reported data for 27 individuals (1 HIV positive and 26 HIV negative) with missing HIV test results in the survey.

**Table 1:** Characteristics of the study population stratified by gender

	Men n = 5124 (38.7%)	Women n = 8128 (61.3%)
Mean age	28.9	31.7
Age group		
15–24	2159 (42.1%)	2622 (32.3%)
25–34	1412 (27.6%)	2359 (29.0%)
35–44	989 (19.3%)	1657 (20.4%)
45–54	564 (11.0%)	1490 (18.3%)
Site type		
Estates	1477 (28.8%)	1995 (24.5%)
Small towns	933 (18.2%)	1399 (17.2%)
Roadside trading center	1009 (19.7%)	1673 (20.6%)
Subsistence farming areas	1705 (33.3%)	3061 (37.7%)
Employed	3257 (63.6%)	2429 (29.9%)
Highest level of education		
None or primary	965 (18.8%)	2822 (34.7%)
Secondary	3991 (77.9%)	5201 (64.0%)
Higher	167 (3.3%)	105 (1.3%)
Relationship status		
Never been in long term relationship	2190 (42.8%)	1378 (17.0%)
Separated or divorced	148 (2.9%)	647 (8.0%)
Widowed	83 (1.6%)	946 (11.7%)
Still in union	2696 (52.7%)	5152 (63.4%)
HIV positive	648 (12.7%)	1484 (18.3%)
STI symptoms	298 (5.8%)	677 (8.3%)

9.6% (1277/13,252) of survey participants had psychological distress as defined by scoring above the cut-off point on the SSQ; 4.5% (231/5124) for males and 12.9% (1046/8128) for females. The mean and median SSQ scores were 1.7 and 1 for men and 2.7 and 2 for women respectively. 25.2% (321/1273) of participants with psychological distress were infected with HIV.

### Risk Factors for Psychological Distress

Individuals with complete data for all variables assessing background, socio-demographic, recreational drug and health-related risk factors for psychological distress were retained (n = 12,748). Table 2 presents the prevalence of psychological distress amongst men and women by characteristic, and the results from the age-adjusted and multivariable risk factor analyses.

**Table 2:** Associations between socio-demographic, behavioural and health characteristics and psychological distress, Manicaland, Zimbabwe, 2009–2011

Characteristic	Men (n = 4878)			Women (n = 7870)		
	No. (%)	Adjusted odds ratio (95% CI)		No. (%)	Adjusted odds ratio (95% CI)	
		Model 1	Model 2		Model 1	Model 2
Socio-demographic characteristics						
Site type						
Small towns	39 (4.5)	0.90 (0.61–1.33)	0.78 (0.38–1.61)	185 (13.5)	1.06 (0.88–1.28)	0.92 (0.64–1.32)
Agricultural estates	72 (5.2)	1.05 (0.75–1.45)	0.97 (0.55–1.73)	229 (12.0)	0.89 (0.75–1.06)	0.80 (0.59–1.05)
Roadside trading centers	31 (3.1)	0.61 (0.41–0.95)	0.78 (0.48–1.28)	203 (12.5)	0.92 (0.76–1.10)	1.16 (0.91–1.47)
Subsistence farming areas	81 (5.0)	1 (Ref)	1 (Ref)	395 (13.4)	1 (Ref)	1 (Ref)
Interview date						
January–March	42 (3.4)	1 (Ref)	1 (Ref)	245 (11.6)	1 (Ref)	1 (Ref)
April–June	64 (5.5)	1.65 (1.11–2.45)	1.54 (0.98–2.44)	292 (13.8)	1.25 (1.04–1.49)	1.33 (1.06-1.69)
July–September	44 (3.9)	1.15 (0.75–1.78)	1.11 (0.60–2.07)	211 (13.0)	1.16 (0.95–1.42)	1.49 (1.09-2.03)
October–December	73 (5.4)	1.63 (1.11–2.41)	1.75 (0.85–3.58)	264 (13.2)	1.22 (1.01–1.47)	1.46 (0.99-2.16)
Age-group						
15–24	93 (4.5)	1 (Ref)	1 (Ref)	207 (8.2)	1 (Ref)	1 (Ref)
25–34	56 (4.2)	0.92 (0.65–1.29)	0.72 (0.49–1.04)	286 (12.5)	1.59 (1.32–1.93)	1.07 (0.87-1.33)
35–44	49 (5.2)	1.17 (0.82–1.67)	0.73 (0.49–1.11)	276 (17.2)	2.33 (1.92–2.82)	1.39 (1.11–1.75)
45–54	25 (4.7)	1.03 (0.66–1.62)	0.65 (0.38–1.10)	243 (16.7)	2.25 (1.84–2.74)	1.08 (0.83–1.40)
Education						
None or primary	48 (5.3)	1 (Ref)	1 (Ref)	487 (17.8)	1 (Ref)	1 (Ref)
Secondary	171 (4.5)	0.87 (0.61–1.23)	0.99 (0.68–1.43)	521 (10.4)	0.66 (0.57–0.77)	0.66 (0.56–0.78)
Higher	4 (2.5)	0.47 (0.17–1.31)	0.59 (0.21–1.71)	4 (3.9)	0.22 (0.08–0.59)	0.29 (0.10–0.81)
Employment status						
Unemployed	75 (4.3)	1 (Ref)	–	816 (14.8)	1 (Ref)	1 (Ref)
Employed	148 (4.7)	1.11 (0.83–1.47)	–	196 (8.4)	0.57 (0.48–0.67)	0.63 (0.53–0.76)
Marital status						
Single	95 (4.5)	1.11 (0.75–1.66)	–	75 (5.7)	0.50 (0.38–0.65)	0.74 (0.56–0.99)
Married	113 (4.4)	1 (Ref)	–	627 (12.5)	1 (Ref)	1 (Ref)
Divorced or separated	9 (6.5)	1.51 (0.75–3.05)	–	119 (19.0)	1.61 (1.29–2.00)	1.69 (1.35–2.13)
Widowed	6 (7.5)	1.70 (0.72–4.02)	–	191 (20.7)	1.59 (1.31–1.93)	1.44 (1.17–1.77)
Religion						
Traditional	12 (15.8)	4.79 (2.48–9.28)	2.53 (1.25–5.14)	9 (31.0)	3.37 (1.51–7.48)	1.74 (0.75–4.06)
Christian	87 (3.7)	1 (Ref)	1 (Ref)	442 (11.2)	1 (Ref)	1 (Ref)
Spiritual	74 (5.9)	1.62 (1.18–2.23)	1.56 (1.12–2.18)	423 (15.7)	1.50 (1.30–1.74)	1.36 (1.17–1.58)
Other	24 (4.13)	1.11 (0.70–1.76)	0.97 (0.60–1.55)	118 (11.2)	1.04 (0.84–1.29)	0.98 (0.78–1.22)
None	26 (4.0)	1.07 (0.68–1.67)	0.63 (0.38–1.03)	20 (14.4)	1.39 (0.85–2.25)	0.78 (0.46–1.33)
Community participation						
Church group						
No	113 (6.6)	1 (Ref)	1 (Ref)	256 (16.9)	1 (Ref)	1 (Ref)
Yes	110 (3.5)	0.51 (0.39–0.67)	0.39 (0.28–0.53)	756 (11.9)	0.79 (0.73–0.85)	0.83 (0.76–0.90)
Social group						
No	124 (4.2)	1 (Ref)	1 (Ref)	340 (11.3)	1 (Ref)	1 (Ref)
Yes	99 (5.2)	1.24 (0.94–1.62)	1.44 (1.09–1.91)	672 (13.8)	1.05 (0.98–1.13)	1.09 (1.01–1.18)
Health-related behaviours						
Alcohol consumption						
No bar visits or <3 drinks	140 (4.2)	1 (Ref)	1 (Ref)	991 (12.7)	1 (Ref)	1 (Ref)
Bar visits and ≥3 drinks	83 (5.4)	1.31 (0.97–1.75)	1.79 (1.29–2.48)	21 (24.1)	1.67 (1.01–2.76)	1.11 (0.63–1.96)
Drugs for pleasure						
No	198 (5.2)	1 (Ref)	1 (Ref)	991 (12.7)	1 (Ref)	1 (Ref)
Yes	25 (2.4)	0.43 (0.28–0.66)	0.35 (0.22–0.55)	21 (41.2)	4.12 (2.33–7.27)	3.74 (2.01–6.93)
Health conditions						
Sexually transmitted infections^a^						
No	195 (4.2)	1 (Ref)	1 (Ref)	817 (11.3)	1 (Ref)	1 (Ref)
Yes	28 (10.0)	2.54 (1.65–3.90)	2.45 (1.56–3.85)	195 (29.6)	3.09 (2.57–3.71)	2.76 (2.28–3.34)
HIV infection						
No	181 (4.3)	1 (Ref)	1 (Ref)	737 (11.5)	1 (Ref)	1 (Ref)
Yes	42 (6.8)	1.66 (1.15–2.40)	1.51 (1.02–2.24)	275 (19.0)	1.63 (1.39–1.90)	1.24 (1.05–1.48)

Model 1 includes adjustment for age. Model 2 includes all variables with p < 0.2 in Model 1

*CI* confidence interval

^a^Sexually transmitted infections based on self-reported symptoms (sores, discharge or pain)

For men, after adjusting for age, elevated risk of psychological distress was associated with HIV infection, reporting other sexually transmitted infections (STI), belonging to traditional or spiritual religions and being interviewed in the immediate post-harvest and early rainy season periods (April to June and October to December, respectively). Men living in roadside trading centers, those participating in church groups and those taking recreational drugs were at reduced risk of psychological distress. HIV infection, other STIs, membership of traditional and spiritual religions, and membership of a non-church-related social group and excessive alcohol consumption remained associated with higher levels of psychological distress in multivariable analysis. Using recreational drugs and membership of a church group remained associated with lower psychological distress.

For women, in the age-adjusted analysis, those with HIV infection, those suffering from other STIs, those who were divorced, separated or widowed, those belonging to traditional or spiritual churches, those consuming excessive amounts of alcohol, those taking recreational drugs, and those interviewed in the immediate post-harvest and early rainy season periods had higher psychological distress. Young age (15–24 years), greater school education, being single (never married), being in a job, and being a member of a church group were associated with lower psychological distress. In the multivariable analysis, all of these associations remained significant with the exception of traditional religion and alcohol consumption. The effect of younger age was weakened, and being interviewed in the dry season (July to September) rather than in the early rainy season was associated with greater psychological distress. Being a member of a non-church-related social group was associated with higher psychological distress.

### Psychological Distress and Sexual Risk Behaviours for HIV Infection

Amongst men not infected with HIV, no associations were observed between psychological distress and having multiple sexual partners in the last 30 days or having multiple current sexual relationships (Table 3). However, psychological distress was associated with transactional sex (AOR = 2.51, 95% CI 1.64–3.84) and with having a partner in a concurrent relationship but not using condoms consistently (AOR = 4.27, 95% CI 1.87–9.76). In uninfected women, psychological distress was associated with engaging in transactional sex (AOR = 4.14, 95% CI 2.16–7.96), engaging in sex work (AOR = 3.57, 95% CI 1.41–9.00), and having a partner in a concurrent relationship but not using condoms consistently (AOR = 2.64; 95% CI 2.20–3.17).

**Table 3:** Associations between psychological distress and sexual risk behaviors for HIV acquisition and HIV transmission in sexually-experienced men and women, Manicaland, Zimbabwe, 2009–2011

	HIV− participants			HIV+ participants		
	No. (%)		AOR (95% CI)	No. (%)		AOR (95% CI)
	Distress 114 (4.0)	No distress 2706 (96.0)		Distress 40 (6.6)	No distress 568 (93.4)	
Men						
Multiple partners in last 30 days	6 (5.3)	144 (5.4)	0.98 (0.42–2.28)	2 (5.0)	27 (4.8)	0.97 (0.22–4.27)
Multiple current sexual relationships	10 (8.8)	189 (7.0)	1.28 (0.66–2.49)	1 (2.5)	38 (6.7)	0.32 (0.04–2.40)
Transactional sex	33 (29.5)	387 (14.4)	2.51 (1.64–3.84)	12 (30.8)	143 (25.4)	1.30 (0.64–2.66)
Partner in concurrent relationship but condom use intermittent	7 (6.1)	41 (1.5)	4.27 (1.87–9.76)	1 (2.5)	8 (1.4)	2.13 (0.25–18.0)

	HIV− participants			HIV+ participants		
	No. (%)		AOR (95% CI)	No. (%)		AOR (95% CI)
	Distress 705 (12.9)	No distress 4778 (87.1)		Distress 275 (19.0)	No distress 1172 (81.0)	
Women						
Multiple partners in last 30 days	1 (0.1)	11 (0.2)	0.63 (0.08–4.90)	3 (1.1)	14 (1.2)	1.01 (0.29–3.57)
Multiple current sexual relationships	5 (0.7)	13 (0.3)	2.77 (0.98–7.82)	2 (0.7)	13 (1.1)	0.72 (0.16–3.23)
Transactional sex	15 (2.1)	24 (0.5)	4.14 (2.16–7.96)	12 (4.4)	40 (3.5)	1.26 (0.64–2.47)
Sex work	7 (1.0)	13 (0.3)	3.57 (1.41–9.00)	9 (3.3)	23 (2.0)	1.68 (0.76–3.70)
Partner in concurrent relationship but condom use intermittent	210 (29.8)	670 (14.0)	2.64 (2.20–3.17)	67 (24.4)	186 (15.9)	1.88 (1.36–2.61)

*CI* confidence interval, *AOR* odds ratios adjusted for age and site type

There was little evidence for associations between psychological distress and sexual risk behaviours for HIV transmission in HIV-infected men and women. However, in HIV-infected women, psychological distress was associated with unprotected sex with partners in concurrent sexual partnerships (AOR = 1.88, 95% CI 1.36–2.61).

### Psychological Distress and the HIV Care Cascade

In HIV-uninfected men, 28.8% (1272/4423) reported ever having had an HIV test; no association was found with psychological distress. In uninfected women, 61.8% (4079/6604) reported having had an HIV test; those with psychological distress were more likely to have had a test but the difference was not statistically significant after adjustment for age and site type (65.4 vs. 61.3%, AOR = 1.14; p = 0.117).

**Fig. 2:**
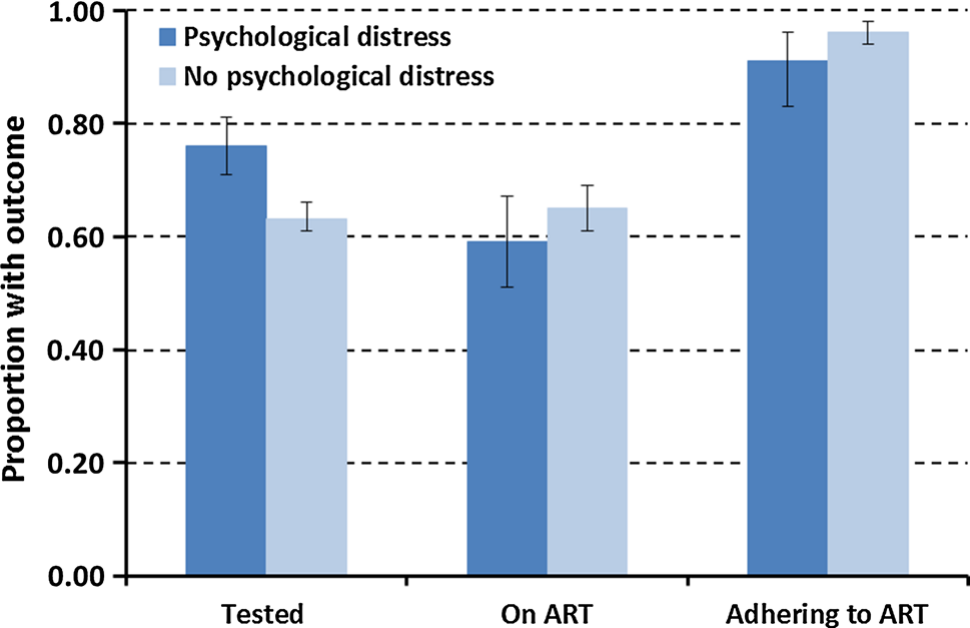
Comparison of HIV care cascades for adults with and without psychological distress in Manicaland, East Zimbabwe, 2009–2011

In HIV-infected men and women who were HIV positive by the study DBS test only (n = 2132), those with psychological distress were more likely to have had an HIV test (75.8%, 241/318) than those without psychological distress (63.4%, 1148/1810; AOR = 1.77, 95% CI 1.34–2.33) (men: 54.8% (23/42); AOR = 1.40 95% CI 0.74–2.65; women: 79.0% (218/276 AOR = 1.46 95% CI 1.06–2.01) (Fig. 2). In addition, men and women with psychological distress were more likely to report cotrimoxazole use (both in those not yet on ART and in those on ART) but the difference was statistically significant after adjusting for age, site type and ART uptake (AOR = 2.31, 95% CI 1.13–4.41).

In HIV-infected men and women who had been tested, knew their infection status, and had data available on ART uptake (n = 723), 63.3% (458/723) had been initiated on ART. No difference in ART uptake was found between those with and those without psychological distress, adjusting for age and site-type (p = 0.125) (Fig. 2).

Overall, self-reported adherence to ART was high amongst HIV-infected individuals who had been initiated on ART (95.4%, 437/458). However, adherence was slightly lower in HIV-infected individuals on ART who suffered from psychological distress than in those without psychological distress [91% (75/82) vs. 96% (361/375), AOR = 0.38; 95% CI 0.15–0.99]. All HIV-infected men with psychological distress reported being adherent to ART (n = 6), while 93.8% (90/96) of those without psychological distress reported being adherent to ART. Amongst women, 91.1% (71/78) of those experiencing psychological distress reported being adherent to ART, as did 95.8% (271/283) of those not suffering from psychological distress (AOR = 0.41; 95% CI 0.15–1.08).

## Discussion

In a general population sample in east Zimbabwe, we found evidence that psychological distress (PD) is more common in PLHIV. Sexual risk behaviours occurred more frequently in uninfected individuals (particularly in women) with PD which suggests that poor mental health may be a risk factor for HIV acquisition. There was little evidence though that PD contributes to high risk behaviour for HIV transmission from previously infected individuals. HIV-infected individuals with psychological distress were more likely to have had an HIV test—in part, perhaps due to worry arising from the knowledge of potentially being infected, especially at more advanced stages of infection. Amongst those who knew their infection status, those with psychological distress were no more likely to have taken up ART. Those on ART with psychological distress, were less likely to be adhering well to their medication.

In previous studies, the average population prevalence of depression has been found to be approximately 5.5% in sub-Saharan Africa with 8% in PLHIV [35]. In this study, we found relatively high levels of psychological distress; 9.6% overall and 15.0% in HIV-infected individuals. Earlier studies in Zimbabwe however, found much higher levels. For example, 51.6% of HIV-infected adolescents aged 15–23 across 3 provinces in rural Zimbabwe, including Manicaland, reported an SSQ score ≥8 while 42% of adults aged 18 and over living in Harare reported an SSQ score >7 [8, 36]. Some of these differences may be accounted for by dissimilarities in measurement including sampling methods, questionnaires and cut-off points used [37]. Differences in the timing of the studies may also be important: the data for the current study were collected between 2009 and 2011, a time of modest economic recovery and rapid scale-up of ART services in Zimbabwe, whereas some of the earlier studies took place in a period of political instability and economic decline which culminated in the collapse of the local currency in February 2009. Our findings of correlations between PD and unemployment are consistent with this interpretation. Other correlates included low educational achievement, being female; and being between the ages of 25–44; results which are consistent with those from other studies in sub-Saharan Africa [38–40].

Our results provide robust scientific evidence from a large, general population survey to support findings of an association between poor mental health and reduced adherence to ART medication from earlier, largely small-scale, clinic-based studies in sub-Saharan Africa [20–25]. An important limitation of the study, however, is its reliance on cross-sectional data. As suggested in the theoretical framework developed from the literature in this study, a number of the key associations for which we found evidence could be due to reverse causation. For example, involvement in sexual behaviour that can result in HIV infection may be a source of psychological distress as well as a possible consequence. Therefore, these results should be interpreted with caution and warrant further investigation in longitudinal studies. While the SSQ is a well validated screening tool for common mental disorders, adapted to fit the local cultural context of Zimbabwe, and populations in neighbouring Shona-speaking countries, it is important to note that the questionnaire was initially developed within a primary care setting in a high-density suburb of Harare, while the present analysis utilises data from a large population based cohort in eastern Zimbabwe. Despite this difference, the use of culturally appropriate idioms in the development of the SSQ, as well as the simplicity of its administration as either a self-completion or an interviewer-administered questionnaire, make it a valuable tool for population based screening for common mental disorders which may be used to improve case finding and linkage to psychiatric care among high risk individuals within the population. Our analysis of associations between psychological distress and ART uptake and adherence was restricted to 875 of the 2132 HIV infected participants (by study’s DBS sero-test) who additionally self-reported being HIV positive. Under-reporting of HIV positive status (e.g. due to fear of stigma) therefore may have biased our results. In addition, approximately 100 individuals reported that they were HIV positive but tested HIV negative in the survey. This indicates that information bias may have occurred through non-differential misclassification of HIV disease status which may have under-estimated the associations measured.

Adherence to ART was assessed through self-reporting and, therefore, is liable to be over-reported [41]. Furthermore, over-reporting may be higher or lower in people suffering from poor mental health. In the study, testing of DBS samples for the presence of Nevirapine (NVP), one component of the most commonly-used ART regimen in Zimbabwe at the time of data collection, was conducted. Amongst individuals who reported being on ART, 77.5% (434/560) had a detectable volume of NVP in their sample, of those, 63.1% (274/434) had a therapeutic concentration [42]. Among 98 individuals with low levels of NVP, 30.6% (30/98) reported having received treatment for Tuberculosis (TB) which may have led to the low NVP concentration [43, 44]. These findings indicate some reliability of self-reported ART adherence; however, in future studies, the full use of biomarker data assessing adherence in conjunction with self-reporting, is recommended [42]. This study was conducted in Manicaland, the second most populous province in Zimbabwe. Given the consistency of the outcomes reported here, with similar research in Sub-Saharan Africa, and the close match between HIV prevalence in Manicaland and national HIV prevalence in Zimbabwe [45], these findings could be generalized across Zimbabwe and potentially other low income countries in the region with similar HIV epidemics.

The results of this study support proposals for greater integration of mental health services with HIV prevention and treatment services in populations suffering from high levels of psychological distress. In east Zimbabwe, we found that psychological distress was highest in women and that, in uninfected women, sexual risk behaviours were more common in those with psychological distress. Furthermore, ART adherence was lower in adults with poor mental health. Therefore, inclusion of screening and counselling for depression within routine antenatal care (ANC) and prevention of mother-to-child transmission (PMTCT) services could be a particularly effective intervention, especially as the WHO has recently suggested an expansion in ART eligibility to include all HIV infected individuals regardless of their CD4 cell count or clinical disease staging [46]. One promising approach is the use of a low cost problem-solving intervention known as the ‘friendship bench’ which was recently developed and piloted in Zimbabwe with the aim of providing care for common mental disorders [8]. The service is run by trained lay health workers within the clinic grounds and is targeted at referred individuals with SSQ scores ≥8. The intervention has been found to reduce SSQ scores amongst participants in Harare, Zimbabwe [8].
